# Environmental Exposure to Cadmium: Health Risk Assessment and its Associations with Hypertension and Impaired Kidney Function

**DOI:** 10.1038/srep29989

**Published:** 2016-07-14

**Authors:** Haiyun Wu, Qilin Liao, Steven N. Chillrud, Qiang Yang, Lei Huang, Jun Bi, Beizhan Yan

**Affiliations:** 1 State Key Laboratory of Pollution Control and Resource Reuse, School of the Environment, Nanjing University, Xianlin Campus, 163 Xianlin Avenue, Nanjing 210023, China; 2 Geological Survey of Jiangsu Province, Nanjing 210018, China; 3 Lamont-Doherty Earth Observatory, Columbia University, Palisades, NY 10964, USA

## Abstract

Cadmium (Cd) is a toxic metal. This study was aimed to estimate the potential health risks in a Cd-polluted district in China, and examine the relationship between urinary cadmium(UCd) and hypertension and impaired kidney function at low exposure levels (UCd: GM 1.3 μg/g creatinine). Blood pressure measurement, questionnaires, and collection of urinary samples were conducted from 217 residents. Environmental samples, food, and cigarette samples were collected and detected to estimate the risks posed by Cd and the contribution of inhalation, ingestion, and dermal contact pathways to these risks. A logistic regression model was used in examining associations between exposure and hypertension and impaired kidney function. Results show that this population is at high risk. For non-smokers, incremental lifetime cancer risk (ILCR) and hazard quotient (HQ) are 1.74E-04 and 2.96, and for smokers, they are 1.07E-03 and 52.5, respectively. Among all exposure pathways, smoking and foods cause the major increases in ILCR and HQ. UCd is significantly associated with hypertension (odds ratio (OR) = 1.468; 95% confidence interval (CI): 1.104, 1.953; *P* = 0.008) and impaired kidney function (OR = 1.902, 95% CI: 1.054, 3.432; *P* = 0.033). The results demonstrate that Cd can potentially lead to adverse health effects.

Cadmium (Cd) is a toxic metal present in food, tobacco smoke, air, water and other media, and it can enter human bodies through inhalation, ingestion and dermal contact (ATSDR 2012). Cd can accumulate in various organs and tissues, but mostly in kidney cortex^1^. In fact, kidney has been considered as the most sensitive target organ for Cd effects^2^.

Studies have evaluated population health risks due to Cd exposure through various pathways, e.g. direct ingestion of water and accidentally soil, consumption of food grown in contaminated fields, inhalation of dust, and dermal contact of soil and water^3^,^4^,^5^. For smokers, tobacco is another significant source of Cd as tobacco plant is able to uptake it from the soil^6^. Assessing collective Cd exposure from all potential pathways is very important. Human health risk assessment has been recognized and widely used as an important tool for assessing risks of developing adverse health outcomes in humans who are exposed to chemicals.

Urinary Cadmium (UCd) has been used as a biomarker to indicate ongoing and chronic Cd exposure level in general population^7^. The World Health Organization (WHO) set a UCd level of 5.24 μg/g creatinine as a threshold^1^, while the threshold set by European Food Safety Authority (EFSA) is 1 μg/g creatinine^1^. The average of UCd in the US population is 0.18 μg/g creatinine (95^th^ percentile: 1.03 μg/g creatinine)^8^.

Cd exposure has been inconsistently associated with blood pressure. Gallagher and Meliker suggested that blood Cd was positively associated with blood pressure among women (N = 5988, U.S., Croatia, and Belgium) but UCd was inversely associated with hypertension (HTN) (N = 8750, U.S., and Japan)^9^. Swaddiwudhipong and Mahasakpan performed a study involved 5,273 subjects in Thailand and concluded that environmental exposure to Cd, indicated by UCd, may increase the risk of hypertension^10^. Thus, the association of UCd and hypertension remains inconclusive and more evidence is required wordwide.

β_2_-microglobulin (BMG) and N-acetyl-β-glucosaminidase (NAG) have been used as renal biomarkers to indicate Cd body burden and its effect on renal function^11^. When UCd is less than 10 μg/g creatinine during exposure, the cadmium-induced impaired kidney function might be reversible after the exposure decreased^12^,^13^. However, if Cd exposure was higher than 10 μg/g, the impair can be irreversible or even deteriorated after the exposure declined^12^,^13^. However, although it has been documented that occupational Cd exposure can cause the tubular effects and glomerular dysfunction, the association between renal effects and environmental exposure in general populations still needs further investigations^12^.

As a fast-developing country, China has been facing an increasingly serious problem of Cd contamination in the environment^14^. In Chenzhou of Hunan province, where the collapse of a tailing dam in a lead and zinc mine led to the spread of mining waste spill on farmland along the Dong River, rice had high Cd concentrations (Arithmetic Mean (AM): 6.99 mg/kg) in its edible parts^15^. In Wenling of Zhejiang province, a well-known electronic and electric waste recycling center, Cd was found at elevated levels (AM: 0.072 ± 0.105 mg/kg) in rice^16^. Many people in China are exposed to elevated Cd exposure^17^,^18^,^19^, which has been reported to be associated with certain adverse health effects^15^,^19^,^20^. A study involving a total of 6103 participants who lived in five Cd polluted areas of China found that, the mean UCd level is 4.82 μg/g creatinine (geometric mean (GM), ranged from 0.08 to 56.99 μg/g creatinine) for males and 4.87 μg/g creatinine (GM, ranged from 0.05 to 57.27 μg/g creatinine) for females^17^. Besides, relatively low environmental cadmium exposure, i.e., UCd < .2.0 μg/g creatinine, is more widespread^21^. Thus, it is vitally important to understand the relationships between Cd exposure and its potential health impacts, especially under low levels.

Our previous study found the soil in a district of WuXi city in eastern China was polluted by Cd (AM: 4.28 mg/kg) and the rice (AM: 0.89 mg/kg) were contaminated^22^. The so-called “Cd-rice” has drawn a lot of attention from the public. The main purpose of this study is to estimate the risk level of human health by taking all potential exposure pathways into consideration in this district. This study also determines the major sources of elevated risk in the studied population, and examines the relationship between UCd and hypertension and the relationship between UCd and impaired kidney function under the low exposure levels.

## Results

### Cd in environmental samples and food samples

The analysis results show that all the tap water and well water samples (n = 6) in the studied area have Cd concentrations below the guideline values^23^,^24^ (Table 1). All the trace heavy metal concentrations meet the national drinking water quality criteria^23^. Most of the water samples have Cd concentrations that are below the detection limit (0.1 μg/L). Cd concentrations in both indoor and outdoor air samples are less than the reference (0.005 μg/m^3^)^25^, so are other trace heavy metals (Table 1).

The mean Cd concentration in 46 soil samples is 4.28 mg/kg, much higher than the guideline value of 0.40 mg/kg (Class 2, agriculture soil). Among 29 home-grown vegetables, canola and crown daisy have high Cd concentrations that exceed the national standards^26^ (Table 1). It is well known that rice can uptake Cd from soil. The mean Cd concentration in 45 home-grown rice is 0.89 mg/kg, suggesting that Cd contamination in the agricultural soil has resulted in Cd accumulation in rice (background value: 0.13 mg/kg for Jiangsu Province^27^). Besides, food produced from other towns, including rice, pork and eggs, vegetables were collected from the local markets, too (Table 1). Cd concentrations in these foods are within safety standards^26^. The mean Cd concentrations in vegetables and rice are 0.017 mg/kg and 0.02 mg/kg, respectively. Cd content in pork and eggs are both lower than the detection limits (0.01 mg/kg). Cigarettes contain high levels of Cd concentration. However, there is no national guideline in China for cigarette currently.

### Characteristics of subjects

A total of 217 residents aged 23 years and older living in the cadmium-contaminated district participated in this investigation. The characteristics of the study population by gender are detailed in Table 2. Of the participants, 80 were males and 137 were females. No significant differences existed between men and women in age, body mass index, urinary cadmium, systolic blood pressure, BMG, NAG, and diabetes. The mean level of diastolic blood pressure for women was significantly lower than that for men (*P-*value < 0.05) and so was the incidence of hypertension (*P-*value < 0.05). The percentage of smokers is significantly different in males and females (*P-*value < 0.01). Of all the males, 67% were smokers while only 3% in females were smokers. Indigenous exposure parameters of the study population, by gender, are provided in Table S2, Supplementary Information.

### ILCR and HQ

The mean ILCR for smokers is 1.07E-03 (5^th^ percentile: 9.69E-04, 95^th^ percentile: 1.17E-03), which means that the probability that an individual may develop cancer when exposed to Cd for a lifetime is 1 in 934, exceeding the common referenced benchmark of 10^−6^–10^−4^ for the protection of public health used by the USEPA^28^. The mean HQ for smokers is 52.5 (5^th^ percentile: 47.1, 95^th^ percentile: 57.8), indicating that there are high non-cancer risks. For non-smokers, the mean ILCR is 1.74E-04 (5^th^ percentile: 1.57E-04, 95^th^ percentile: 1.93E-04) and the mean HQ is 2.96 (5^th^ percentile: 2.73, 95^th^ percentile: 3.20), both higher than thresholds.

**Figure 1:**
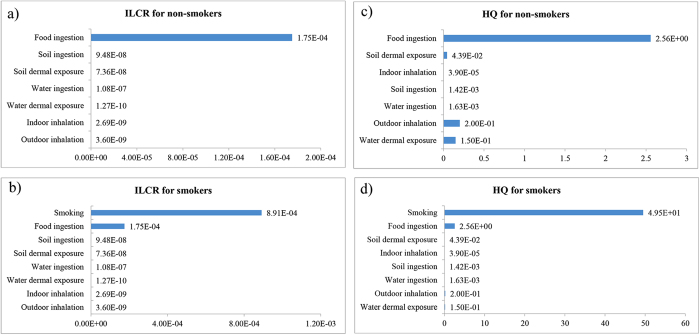
Multi-pathway analysis of ILCRs and HQs in non-smokers and smokers. (**a**) ILCR for non-smokers. (**b)** ILCR for smokers. (**c)** HQ for non-smokers. (**d**) HQ for smokers.

Multi-pathway analysis of ILCR and HQ in non-smokers and smokers is shown in Fig. 1. For non-smokers, 7 pathways are allowed for, including food, soil, and water ingestion, indoor and outdoor inhalation, soil and water dermal contact. The exposure to and uptake of Cd was mainly from food ingestion, which contributed 86.6% to the total HQ and 99.8% to the total ILCR. Outdoor inhalation and water dermal contact are also important contributions to total HQ. For smokers, while food ingestion remains an important pathway that accounts for 5.0% of the lifetime cancer risk, smoking is the most important source for ILCR (94.4%). However, the alarming truth is that the HQ has increased to 52.5 due to the additional exposure through smoking. Food ingestion alone contributes 16.4% to the non-cancer hazard, the HQ being 2.56. Actually, home-grown rice, of which Cd content has exceeded the Chinese criterion, is the main source of food Cd ingestion (see Table 1).

### Urinary cadmium

The geometric mean (GM) UCd concentration of the subjects was 1.26 μg/g creatinine (Table 2), which is within the threshold set by the WHO (5.24 μg/g creatinine) but beyond the one set by the EFSA (1 μg/g creatinine)^1^. Of all the participants, 65% had UCd concentration that exceeded the value of 1 μg/g creatinine. For both genders, the average UCd concentrations in non-smokers were lower than those in smokers (Table 3). Subjects who consumed home-grown rice had higher UCd concentrations (GM 1.30 μg/g creatinine) than those who ate rice bought from the market (GM 1.21 μg/g creatinine).

### Blood pressure and hypertension

**Figure 2:**
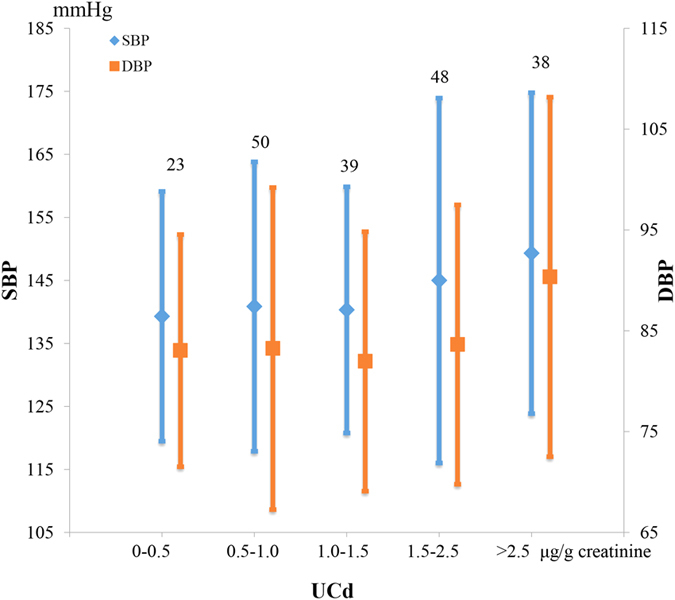
Mean SBP and DBP by UCd level. The suspending figures represent subject numbers of each group. The error bars represent the standard deviations.

UCd was classified into five levels based on the cut-off level of 0.5, 1.0, 1.5, and 2.5 μg/g creatinine. As shown in Fig. 2, SBP and DBP both increase with UCd generally. The dramatic shift occurs in group 5 (UCd above 2.5 μg/g creatinine). Except for the slightly decline in DBP when UCd level is 1.0–1.5 μg/g creatinine, blood pressure increases with UCd level, especially when UCd > 2.5 μg/g creatinine.

Table 4 presents the results of the logistic regression analysis of the determinants of hypertension. To avoid potential interference, subjects who have impaired kidney function defined as BMG > 890 μg/g creatinine or NAG > 9.8 U/g creatinine were excluded in this analysis. Age and body mass index (BMI) are significant factors of HTN, the odds ratios (ORs) being 1.048 (95% confidence interval (CI): 1.018, 1.078; *P* = 0.001), 1.269 (95% CI: 1.125, 1.432; *P* = 0.000) for 166 subjects and 1.055 (95% CI: 1.020, 1.090; *P* = 0.002), 1.255 (95% CI: 1.088, 1.448; *P* = 0.002) for 116 non-smokers. After adjusting for age, BMI, smoking status, and diabetes, the prevalence of hypertension significantly increased with increasing urinary cadmium in total subjects, the OR being 1.468 (95% CI: 1.104, 1.953; *P* = 0.008). The OR is higher (1.601, 95% CI: 1.123, 2.284; *P* = 0.009) when only non-smokers are included. However, no significant association was found among smokers (all men, *P* = 0.746). When the variables were stratified by gender, the OR of HTN is significant in female non-smokers (1.545, 95% CI: 1.082, 2.206; *P* = 0.017), but not in male non-smokers (*P* = 0.239), which might indicate that females are more vulnerable to HTN due to Cd exposure.

### Impaired kidney function

According to the cut-off values of BMG (890 μg/g creatinine) and NAG (9.8 U/g creatinine), impaired kidney function was defined. A total of 29 out of 178 (16.3%) cases of impaired kidney function was found. Table 5 presents the results of the logistic regression analysis of the factors of impaired kidney function. To avoid potential interference, only subjects without hypertension were included. Sex, age, BMI, smoking, and diabetes were also adjusted in addition to urinary cadmium. UCd is significantly related to impaired kidney function in a positive way (*P* = 0.028). The adjusted OR for increasing prevalence of impaired kidney function associated with 1 μg/g creatinine’s increase in urinary cadmium is 1.902 (95% CI: 1.054, 3.432). BMI is also significantly associated with impaired kidney function, with the OR of 1.365 (95% CI: 1.085, 1.719; *P* = 0.008). Age is boderline associated with impaired kidney function (*P* = 0.074). The analysis result suggests that smoking is significantly negatively associated with impaired kidney function (*P* = 0.038). When smokers were excluded, the relationship between UCd and impaired kidney function was shown in Table 5.

## Discussion

Of all the collected environmental and food samples, soil, home-grown rice and some vegetables were found to be contaminated with Cd (see Table 1). Cd accumulation in rice has been well established^29^. A previous study which was conducted in China as well suggested that the consumption of Cd-rice grown in a highly polluted area in Zhejiang Province was the most important determinant of Cd exposure^30^. In the present study, rice consumption is indeed one of the most important pathways of Cd exposure. For non-smokers, food ingestion (including rice and some vegetable ingestion) is the main source compared to soil ingestion and dermal exposure, water ingestion and dermal exposure, indoor and outdoor inhalation. When Cd exposure through smoking is taken into consideration, food ingestion remains the most important pathway for ILCR but not for HQ. For smokers, the HQ has risen from 2.96 up to 52.5, indicating that Cd exposure through cigarette smoking has created larger threats than food ingestion or any other pathways do to human health in this studied area.

The average internal exposure of Cd, determined from urine, was 1.26 μg/g creatinine. This is not a high UCd level compared to other reporting^10^,^13^,^31^. But based on the threshold of 1 μg/g creatinine set by the EFSA, 65% of the subjects had UCd that exceeded the threshold. For both genders, smokers had higher UCd concentrations than non-smokers, which is already well-known^7^. However, the discrepancy was not huge (10.1% and 19.2%) in this study. It has been reported that exposure to secondhand smoke is associated with blood cadmium^32^. Combining the questionnaire-survey we conducted in this area, we deduce that the small discrepancy might be connected with the serious secondhand smoking status.

After adjusting for sex, age, BMI, smoking status, and diabetes, the prevalence of HTN is significantly and positively associated with urinary cadmium in total subjects (OR = 1.468, 95% CI: 1.104, 1.953; *P* = 0.008). When only non-smokers are included, the OR is 1.601 (95% CI: 1.123, 2.284; *P* = 0.009), which is higher than that for a Thai population (OR = 1.043 for males and 1.055 for females) even though the UCd level in this study is lower^10^, and the diet and BMI might lead to the difference. When the population was stratified by sex, this relationship remains significant for females (OR = 1.545, 95% CI: 1.082, 2.206; *P* = 0.017) but not for males. This result is in accordance with previous findings^10^,^33^.

After adjusting for sex, age, BMI, smoking status, and diabetes, UCd is significantly related to impaired kidney function in a positive way among non-hypertensive subjects (OR = 1.902, 95% CI: 1.054, 3.432; *P* = 0.033). Kidney damage at the same Cd exposure level was also revealed by an OSCAR study, which disclosed an increased prevalence of 10% tubular proteinuria (accounting for a normal background prevalence of 5%) at a urinary cadmium concentration of 1.0 nmol/mmol creatinine (0.85 μg/g creatinine)^34^. Smoking is well known as a risk factor for chronic kidney diseases^35^,^36^, which is why we included it as a covariate in the regression analysis. However, the result indicated that smoking status was negatively associated with impaired kidney function (see Table 5). This provocative result indicates more research is needed in the future. One possible reason is that most participants in this study have been substantially exposed to secondhand smoke of cigarette, which can be an important confounder for the effect of smoking status.

Urine Pb level of the population in this study is 3.7 μg/L, which is around the background value of Chinese population^37^ and far below the Chinese diagnostic criteria of 70 μg/L for occupational chronic poisoning (GBZ37-2002). In our screening step of regression analysis, urine Pb had been ruled out because of its insignificance. However, there might be a third possibility of reverse causation for these associations found in this study, i.e., osteoporosis after menopause for old women in this study. Calcium deficiency, following calcium loss in the urine, has been related to the increase in the absorption cadmium^38^,^39^.

In this study, we find that smoking increases Cd exposure, and UCd is significantly positively associated with both hypertension and impaired kidney function. This is consistent with the elevated HQ of Cd exposure in smokers that we have estimated. Cigarette controlling in Cd exposed population should be more heavily publicized in addition to the strict monitoring and excluding of Cd-rice by the government. The limit of 5 μg/g creatinine for UCd in China^40^ should be reconsidered thoroughly.

Our study has two strengths. Firstly, this is a study that takes multi-pathways into consideration when measuring exposure dose. They are food, water and soil ingestion, soil and water dermal exposure, indoor and outdoor exposure, and smoking. Secondly, this study investigated smoking exposure data on individual level. We collected filters, ashes, main body of cigarettes before and after smoked by local smokers to obtain accurate and local exposure dose.

The limitations of this study include, the small sample size of the population and the narrow age range, and the cross-sectional nature of the study. Further longitudinal studies may be useful to infer the relationships between cadmium exposure and adverse health effects.

## Materials and Methods

### Study area and subjects

The studied area is located in Wuxi city, Jiangsu Province, China (see Fig. S1, Supplementary Information). Our previous study has found that soil in the study area is heavily polluted by Cd (AM: 4.28 mg/kg, background value: 0.11 mg/kg), most likely related to its ceramic industry^22^. Residents who has lived here for at least 5 years were recruited in this study (N = 217). Study protocols were reviewed and approved by Nanjing University and informed consents were obtained from all participants. The study was conducted in accordance with the approved guidelines. Each participant was interviewed about demographic characteristics, dietary behaviors, daily activities, smoking habits and medical history of renal disease, hypertension, and diabetes. Height and weight were measured to obtain body mass index (BMI). Blood pressure was measured twice in a sitting position after 5 min rest, and the average was recorded. Technicians who performed the measurement were all trained by physicians.

### Sampling and Analysis

Tap water (n = 3) and well water (n = 3) samples were collected in acid-washed 250-mL polyethylene bottles, and then detected with inductively coupled plasma-mass spectrometry (ICP-MS) using the USEPA 200.8 method. The limit of detection was 0.1 μg/L. Method blank was below detection limit. The recovery rate ranged from 89% to 100%. The relative percent difference of duplicate measurement was less than 6%. Those samples with Cd concentration below the detection limit were treated as 0.005 mg/kg in statistic processing.

Indoor (n = 2) and outdoor air (n = 2) samples were obtained using a PM_10_ air sampler (Qingdao Laoshan Electronic Instrument General Factory Co., Ltd., Qingdao, China) with pre-heated micro quartz fiber paper (MK 360, Munktell Filter AB), and then detected with ICP-MS using the USEPA 2003 method. The detection limit was 0.005 μg. Method blank was below detection limit. The recovery ranged from 102% to 113%. The relative percent difference of duplicate measurement was less than 5%.

Soil samples (n = 46) were taken from the upper 5 cm of ground and stored in clean polyethylene bags and air dried to a constant weight. After the samples were sieved and digested, Cd concentrations were determined using ICP-MS. The detection limit was 0.01 mg/kg. Method blank was below detection limit. Chinese soil geochemical standard reference samples (GBW07427, GBW07428, GBW07429, GBW07430) were used. The recovery ranged from 92% to 107%. The relative percent difference of duplicate measurement was less than 5%.

Raw home-grown and market-bought food samples (n = 29) were collected and stored in polyethylene bags. Home-grown samples include canola, crown daisy, lettuces, spinach, Chinese chive, Chinese cabbages, and rice. Market-purchased samples include crown daisy, Chinese cabbages, amaranth, celery, true squash, rice, pork, and eggs. The samples were washed and then cut into small pieces. After drying to constant weight at 80 °C in an oven, the samples were ground and sieved for acid digestion. Using the USEPA 6020A method, the concentrations of Cd in the samples were determined with ICP-MS. The detection limit was 0.01 mg/kg. Method blank was below detection limit. Element Standard GSB0417212004 was used (National Center of Analysis and Testing for Nonferrous Metals and Electronic Materials (NCATN), Beijing, China). The recovery ranged from 94% to 105%. The relative percent difference of duplicate measurement was less than 5%.

Cigarette samples (n = 6) were collected by inviting 6 indigenous participants to smoke cigarettes of different brands bought from local shops. Filters, ashes, main body of cigarettes were collected before and after smoking, digested and then detected with ICP-MS using the USEPA 6020A method. The detection limit was 0.01 mg/kg. Method blank was below detection limit. Element Standard GSB0417212004 was used (NCATN, Beijing, China). The recovery ranged from 91% to 96%. The relative percent difference of duplicate measurement was less than 9%.

Morning urine samples were collected in acid-washed polyethylene bottles and transported to Nanjing University and stored at −80°C until analysis. Concentration of Cd was detected with ICP-MS. The limit of detection was 0.02 μg/L. No subjects had values below the detection limit. The method precision, calculated as the coefficient of variation for duplicate measurement was 5.0% for UCd. The Seronorm Trace Element Human Urine (Nycomed Pharma AS, Oslo, Norway) was used. The recovery ranged from 89% to 95%. BMG in urine were determined using Latex enhanced immuno-turbidimetry method and NAG was determined using Colorimetry Method using test kits (GS331S, GS341S, Beijing Strong Biotechnologies Co., Ltd., Beijing, China). Urinary creatinine concentration by the Jaffe reaction method was used to adjust for all urine parameters. All these bioanalyses were performed with automatic biochemical analyzer (7180, Hitachi).

### Statistical analyses

Impaired kidney function was defined based on the cut-off values for BMG and NAG of 890 μg/g creatinine and 9.8 U/g creatinine referring to a previous Chinese study^13^. Levels of BMG and NAG above the cut points were regarded as “elevated”, otherwise, “normal”^13^.

We define HTN as mean systolic blood pressure (SBP) ≥140 mmHg, mean diastolic blood pressure (DBP) ≥90 mmHg, self reported physician diagnosis, or medication use^41^.

Binary logistic regression was used to examine the relation between hypertension (dependent variable) and urinary cadmium (independent and continuous variable) and between impaired kidney function (dependent variable) and urinary cadmium (independent and continuous variable), after adjusting for sex, age, BMI, smoking status, and diabetes (stepwise regression was used to select variables, and UCd, sex, age, BMI entered the regression equation, smoking status was included because it was found to be related to hypertension and renal effects^42^,^43^, and diabetes was included because it was reported to be related to Cd and chronic kidney disease^44^,^45^. All statistical analyses were performed using SPSS (Version 22). A two-sided *P* < 0.05 was considered to indicate statistical significance.

### Human health risk assessment

#### Average daily intake (ADI)

The average daily intake of Cd by subjects was calculated using the following equation recommended by the U.S. Environmental Protection Agency (EPA)^28^.


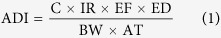


where ADI is the average daily intake or dose through ingestion or inhalation (mg/kg-d); C is the chemical concentration in the exposure medium (mg/L, mg/kg, or mg/m^3^); IR is the ingestion rate (L/d, kg/d, or m^3^/d); EF is the exposure frequency (d/year); ED is the exposure duration (year); BW is the body weight (kg) and AT is the time period over which the dose is averaged (day).

For exposure dose through dermal contact calculation, ADI_D_ was calculated using the following equation.


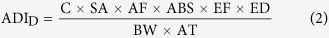


where SA is the exposed skin surface area (cm^2^), AF is the adherence factor (mg/cm^2^/d), and ABS is the dermal absorption factor.

#### Cancer risk: incremental lifetime cancer risk (ILCR)

ILCR is calculated by multiplying the ADI by the cancer slope factor (SF, kg·d/mg, or m^3^/μg)^28^.


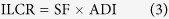


#### Non-cancer hazard: Hazard quotient (HQ)

Non-cancer hazard was assessed by comparing the average daily intake (ADI) to the reference dose (RfD) or the reference concentration in air (RfC) for an individual pathway and chemical.

The values of these parameters were obtained from the above questionnaire-based exposure survey and open database and literature^46^,^47^,^48^,^49^,^50^,^51^(see Supplementary Information Tables S1 and S2).

To account for variability in ADI values and to provide a more meaningful estimate of the probable range of exposures, a probabilistic approach using Monte Carlo simulation was incorporated to provide distributions around all exposure inputs. The simulation was run in Crystal Ball (Oracle Corporation, Vallejo, US) with 10,000 iterations to ensure the stability of the output distributions.

## Additional Information

**How to cite this article**: Wu, H. *et al.* Environmental Exposure to Cadmium: Health Risk Assessment and its Associations with Hypertension and Impaired Kidney Function. *Sci. Rep.* **6**, 29989; doi: 10.1038/srep29989 (2016).

## Supplementary Material

Supplementary Information

## Figures and Tables

**Table 1:** Cd concentrations in environmental media and foods.

Media	Cd concentration	Unit	Guidelines (Chinese)
Tap water	0.1	μg/L	5 (GB 5749-2006)^23^
Well water	0.1	μg/L	10 (GB/T 14848-93, Category III)^24^
Indoor air	0.0015	μg/m^3^	0.005 (GB 3095-2012, Reference)^25^
Outdoor air	0.002	μg/m^3^	0.005 (GB 3095-2012, Reference)^25^
Soil	4.28	mg/kg	0.40 (GB 15618-2008, Class 2, agriculture soil)^52^
Home-grown			
Vegetables	0.55	mg/kg	0.05 (GB 2762-2012)^26^
Rice	0.89	mg/kg	0.2 (GB 2762-2012)^26^
Market-bought			
Vegetables	0.02	mg/kg	0.05 (GB 2762-2012)^26^
Rice	0.02	mg/kg	0.2 (GB 2762-2012)^26^
Pork	<0.01	mg/kg	0.1 (GB 2762-2012)^26^
Eggs	<0.01	mg/kg	0.05 (GB 2762-2012)^26^
Cigarettes	3.56	mg/kg	—

**Table 2:** Characteristics of the study population, by gender.

Characteristics	Males	Females	Total	*P*-value^a^
Number	80	137	217	
Age (years)				
AM ± ASD^b^	59.4 ± 14.0	60.1 ± 15.7	59.9 ± 15.1	0.745
BMI (kg/m^2^)				
AM ± ASD	24.3 ± 3.4	24.5 ± 3.3	24.4 ± 3.3	0.675
Smokers(%)	67	3	27	0.000
Urinary Cd (μg/g creatinine)				
GM (GSD)^c^	1.25 (2.08)	1.27 (2.30)	1.26 (2.21)	0.387
SBP				
AM ± ASD	145.6 ± 21.5	141.9 ± 25.2	143.3 (23.9)	0.277
DBP				
AM ± ASD	87.5 ± 13.0	82.8 ± 15.4	84.5 ± 14.7	0.024
HTN (%)	50	36	41	0.040
BMG (μg/g creatinine)				
GM (GSD)	124.7 (4.6)	106.4 (3.8)	113.4(4.1)	0.163
NAG (U/g creatinine)				
GM (GSD)	3.9 (2.3)	3.7 (2.3)	3.7 (2.3)	0.493
Diabetes (%)	3	3	3	0.856

^a^Difference between males and females.

^b^Arithmetic mean ± arithmetic standard deviation.

^c^Geometric mean (geometric standard deviation).

**Table 3:** Urinary Cadmium concentrations by sex, smoking, and source of rice (μg/g creatinine).

Characteristics	Number	GM^a^	GSD^b^	Range
Sex				
Male	75	1.25	2.08	0.13–4.68
Female	123	1.27	2.30	0.06-13.07
Non-smokers				
Male	25	1.16	2.02	0.31–3.72
Female	119	1.26	2.31	0.06–13.07
Smokers				
Male	50	1.29	2.12	0.13–4.68
Female	4	1.56	1.98	0.63–2.80
Source of rice				
Market	60	1.21	2.00	0.15–4.66
Home-grown	122	1.30	2.21	0.06–13.07
Both	10	0.78	3.51	0.13–7.22

^a^Geometric mean.

^b^Geometric standard deviation.

**Table 4:** Logistic regression analysis of HTN factors.

Subjects	Variables	OR (95% CI)	*P*-value
Total n = 166	UCd (μg/g creatinine)	1.468 (1.104 1.953)	0.008
	Sex (female or male)	0.181 (0.106, 0.531)	0.002
	Age (years)	1.048 (1.018, 1.078)	0.001
	BMI (kg/m^2^)	1.269 (1.125, 1.432)	0.000
	Smoking status (yes or no)	0.377(0.128, 1.114)	0.078
	Diabetes (yes or no)	6.332 (0.438, 91.483)	0.176
Non-smokers n = 116	UCd (μg/g creatinine)	1.601 (1.123, 2.284)	0.009
	Sex (female or male)	0.253 (0.077, 0.827)	0.023
	Age (years)	1.055 (1.020, 1.090)	0.002
	BMI (kg/m^2^)	1.255 (1.088, 1.448)	0.002
	Diabetes (yes or no)	5.751 (0.393, 84.100)	0.201
Smokers n = 50	UCd (μg/g creatinine)	1.096 (0.629, 1.910)	0.746
	Age (years)	1.011 (0.955, 1.069)	0.711
	BMI (kg/m^2^)	1.175 (0.968, 1.426)	0.103
Female nonsmokers n = 97	UCd (μg/g creatinine)	1.545 (1.082 2.206)	0.017
	Age (years)	1.046 (1.009, 1.084)	0.014
	BMI (kg/m^2^)	1.227 (1.054, 1.428)	0.008
	Diabetes (yes or no)	5.744 (0.430, 76.733)	0.186
Male nonsmokers n = 19	UCd (μg/g creatinine)	3.817 (0.411, 35.474)	0.239
	Age (years)	1.109 (1.000, 1.229)	0.049
	BMI (kg/m^2^)	1.632 (0.917, 2.903)	0.096

(Subjects who have impaired kidney function defined as BMG > 890 μg/g creatinine or NAG > 9.0038 U/g creatinine were excluded).

**Table 5:** Logistic regression analysis of impaired kidney function factors.

Subjects	Variables	OR (95% CI)	*P*-Value
Total n = 100	UCd (μg/g creatinine)	1.902 (1.054, 3.432)	0.033
	Sex (female or male)	0.212 (0.031, 1.438)	0.112
	Age (years)	1.050 (0.995, 1.109)	0.074
	BMI (kg/m^2^)	1.365 (1.085, 1.719)	0.008
	Smoking status (yes or no)	0.040 (0.002, 0.839)	0.038
	Diabetes (yes or no)	9.853 (0.351, 276.967)	0.179
Nonsmokers n = 75	UCd (μg/g creatinine)	1.786 (0.978, 3.262)	0.049
	Sex (female or male)	0.242 (0.037, 1.586)	0.139
	Age (years)	1.054 (1.000, 1.111)	0.048
	BMI (kg/m^2^)	1.324 (1.054, 1.662)	0.016

(Only non-hypertensive subjects are included).
