# Supplementary material for: Environmental Exposure to Cadmium: Health Risk Assessment and its Associations with Hypertension and Impaired Kidney Function
Source: Sci Rep. 2016 Jul 14;6:29989. doi: 10.1038/srep29989 (PMC4944124; doi:10.1038/srep29989)
Supplement: Supplementary Information [file srep29989-s1.pdf]

Supplementary Information

Environmental Exposure to Cadmium: Health Risk Assessment and its Associations with Hypertension and Impaired Kidney Function

Haiyun Wu, Qilin Liao, Steven N. Chillrud, Qiang Yang, Lei Huang, Jun Bi*, Beizhan Yan*

Total pages 8

Supplement to exposure assessment Page S2-S4

Tables

Table S1. Risk assessment parameters. Page S5

Table S2. Indigenous exposure parameters of the study population, by gender. Page S6

Figures

Figure S1. Location of study area. This sketch map was generated according to its shape in Baidu Map, using Arcgis 10.1 (http://downloads.esri.com/support/downloads/other_/ArcGIS101sp1-issues.htm). Page S7

Supplement to exposure assessment

A. Dermal exposure

1. Soil dermal exposure

$$ADI_{der\text{-}s} = \frac{C \times CF \times SA \times AF \times ABS \times EF \times ED}{BW \times AT}$$

where, $ADI_{der\text{-}s}$ is the average daily intake through soil dermal exposure (mg/kg-d), C is the Cd concentration in soil (mg/kg), CF is the convert factor (10^{-6} kg/mg), SA is the exposed skin surface area (cm^2), AF is the adherence factor (mg/cm^2); ABS is the dermal absorption factor (unitless), EF is the exposure frequency (d/a), ED is the exposure duration (a), BW is the body weight (kg), and AT is the time period over which the dose is averaged (d).

2. Water dermal exposure

$$ADI_{der\text{-}w} = \frac{C \times SA \times PC \times CF \times ET \times EF \times ED}{BW \times AT}$$

where, $ADI_{der\text{-}w}$ is the average daily intake through water dermal exposure (mg/kg-d), C is the Cd concentration in water (mg/L), SA is the exposed skin surface area (cm^2), PC is the permeability constant (cm/h), ET is the exposure time (h), CF is the convert factor (1 L/1000 cm^3), EF is the exposure frequency (d/a), ED is the exposure duration (a), BW is the body weight (kg), and AT is the time period over which the dose is averaged (d).

B. Ingestion exposure

1. Soil ingestion exposure

$$ADI_{ing\text{-}s} = \frac{C \times IR \times CF \times EF \times ED}{BW \times AT}$$

where, $ADI_{ing\text{-}s}$ is the average daily intake through soil ingestion (mg/kg-d), C is

the Cd concentration in soil (mg/kg), IR is the ingestion rate (mg/d), CF is the convert factor (10^{-6} kg/mg), EF is the exposure frequency (d/a), ED is the exposure duration (a), BW is the body weight (kg), and AT is the time period over which the dose is averaged (d).

2. Water ingestion exposure

$$ADI_{ing\text{-}w} = \frac{C \times IR \times EF \times ED}{BW \times AT}$$

where, $ADI_{ing\text{-}w}$ is the average daily intake through water ingestion (mg/kg-d), C is the Cd concentration in water (mg/L), IR is the ingestion rate (L/d), EF is the exposure frequency (d/a), ED is the exposure duration (a), BW is the body weight (kg), and AT is the time period over which the dose is averaged (d).

3. Food ingestion exposure

$$ADI_{ing\text{-}f} = \frac{\sum_{i=1}^{n} C_i \times IR_i \times CF \times EF \times ED}{BW \times AT}$$

where, $ADI_{ing\text{-}f}$ is the average daily intake through food ingestion (mg/kg-d), C_i is the Cd concentration in food i (mg/kg), IR_i is the ingestion rate of food i (mg/d), CF is the convert factor (10^{-6} kg/mg), EF is the exposure frequency (d/a), ED is the exposure duration (a), BW is the body weight (kg), and AT is the time period over which the dose is averaged (d).

C. Inhalation exposure

$$ADI_{inh} = \frac{C \times IR \times EF \times ED}{BW \times AT}$$

where, ADI_{inh} is the average daily intake through inhalation (mg/kg-d), C is the Cd concentration in air (mg/m^3), IR is the inhalation rate (m^3/d), EF is the exposure frequency (d/a), ED is the exposure duration (a), BW is the body weight (kg), and

AT is the time period over which the dose is averaged (d).

D. Cigarette smoking exposure[1,2]

$$ADI_{smoking} = \frac{\left[(C_m \times M_m - C_a \times M_a) \times \frac{2}{3} - (C_{fa} \times M_{fa} - C_{fb} \times M_{fb})\right] \times \frac{1}{2} \times DTC \times EF \times ED}{BR \times BW \times AT}$$

where, $ADI_{smoking}$ is the average daily intake through smoking (μg/m^3), C_m is the Cd concentration in the main body of cigarettes (mg/kg), M_m is the main body mass (kg/piece), C_a is the Cd concentration in the ashes (mg/kg), M_a is the ash mass (kg/piece), C_{fa} is the Cd concentration in the filters after smoking (mg/kg), M_{fa} is the filter mass after smoking (kg/piece), C_{fb} is the Cd concentration in the filters before smoking (mg/kg), $M_{fb}$is the filter mass before smoking (kg/piece), $\frac{2}{3}$ is the rate of smoking, the rest $\frac{1}{3}$ is spontaneous combusted, $\frac{1}{2}$ is the absorption rate of smog, the rest $\frac{1}{2}$ is exhaled, DTC is the daily cigarette consumption (piece/d), BR is the daily breathing rate, EF is the exposure frequency (d/a), ED is the exposure duration (a), BW is the body weight (kg), and AT is the time period over which the dose is averaged (d).

For smoking, $HQ_{smoking} = \frac{ADI_{smoking}}{MRL}$, where, MRL is the chronic minimal risk level (1.0E-02 μg/m^3)[3].

Indigenous exposure factors, including sleep time every day, outdoor time every day, soil-touching time every day, face wash time every day, bath time every day, hand-laundering time every day, water intake every day, smoking habits, rice and vegetables intake every day, were obtained from the questionnaire survey, while other exposure factors were obtained from Exposure Factors Handbook of Chinese Population (Adults)[4].

Table S1. Risk assessment parameters.

**Parameters**	**Value**	**Source**
**PC** [a]	0.001 cm/h	(Canada, 2004)[5]
**ABS** [b]	0.14	(Canada, 2004)[5]
**SA** [c]	1.7, 1.7, 1.6, 1.6 cm^2 for males who are 18-44, 45-59, 60-79, ≥80; 1.5, 1.6, 1.5, 1.4 cm^2 for females who are 18-44, 45-59, 60-79, ≥80	(MEP, 2013)[4]
**AF** [d]	0.0314 mg/cm^2 for face; 0.1336 mg/cm^2 for hand	(USEPA, 2011)[6]
**IR** [e]	18.4, 18.3, 14.3, 12.4 m^3/d for males who are 18-44, 45-59, 60-79, ≥80; 14.6, 14.9, 13.3, 11.7 m^3/d for females who are 18-44, 45-59, 60-79, ≥80	(MEP, 2013)[4]
**IR_s** [f]	50 mg/d	(USEPA, 2011)[6]
**RfD_{oral}** [g]	1.0E-03 mg/kg-d	(USEPA, 2004)[7]
**RfC** [h]	1.0E-05 mg/m^3	(ATSDR, 2012)[3]
**RfD_{der}** [i]	5.0E-05 mg/kg-d	(USEPA, 2004)[7]
**SF_{inh}** [j]	1.8E-03 m^3/μg	(USEPA, 1992)[8]
**CPF** [k]	6.1 kg-d/mg	(YU, 2014)[9]

[a] Permeability constant; [b] Dermal absorption factor; [c] Exposed skin surface area; [d] Adherence factor; [e] Inhalation rate; [f] Soil ingestion rate; [g] Reference dose for oral exposure; [h] Reference concentration in air; [i] Reference dose for oral exposure; [j] Slope factor for inhalation; [k] Carcinogenic potency factor.

Table S2 Indigenous exposure parameters of the study population, by gender.

Items	Males	Females	Total
**Daily sleep time (h)**	7.2	7.2	7.2
**Daily activity time (h)**	4.2	2.8	3.3
**Soil exposure time (h)**	1.9	1.0	1.4
**Daily face-washing frequency (times)**	2.3	2.4	2.4
**Weekly bath frequency (times)**	2.0	2.4	2.2
**Daily hand-wash clothes time (h)**	0.1	0.3	0.2
**Daily water intake (ml)**	1424	787	1034
**Daily rice intake (g/d)**	286.8	235	255
**Daily vegetable intake (g/d)**	444.4	338	379

Figure S1 Location of study area. This sketch map was generated according to its shape in Baidu Map, using Arcgis 10.1 (http://downloads.esri.com/support/downloads/other_/ArcGIS101sp1-issues.htm).

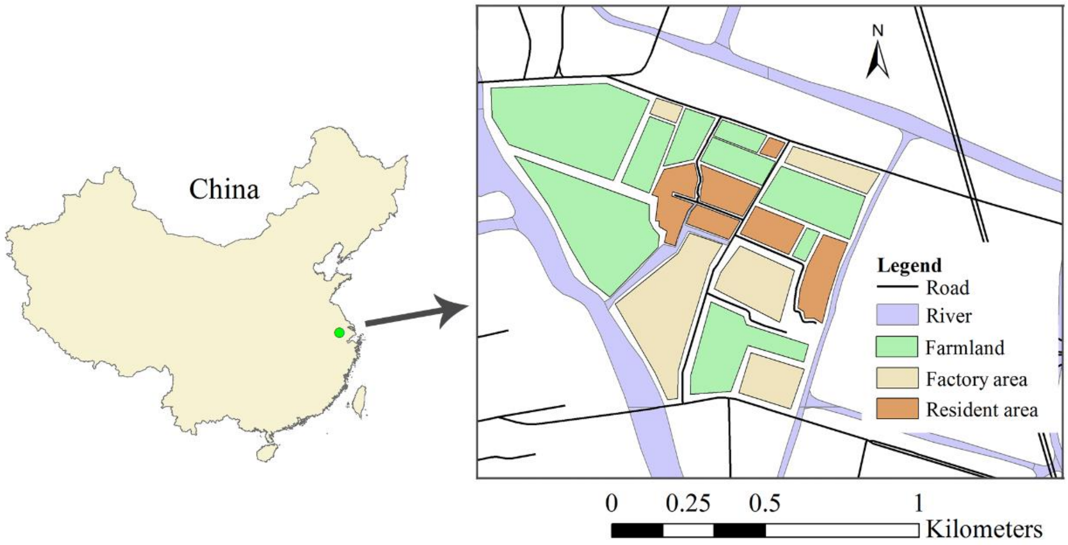

Reference

1 Cui, D. S. & Liu, X. X. ICP-MS determination of 39 elements in cigarettes, ashes, and filters and exploration of elements intake in smokers. *Studies of Trace Elements and Health* **26,** 53-57 (2009).

2 Fowles, J. & Dybing, E. Application of toxicological risk assessment principles to the chemical constituents of cigarette smoke. *Tob Control* **12,** 424-430 (2003).

3 Agency for Toxic Substances and Disease Registry. *Toxicological profile for cadmium*. (2012) Available at: http://www.atsdr.cdc.gov/toxprofiles/tp.asp?id=48&tid=15. (Accessed: 2015).

4 Ministry of Environmental Protection. *Exposure factors handbook of Chinese population (Adults)*. (China Environmental Press, 2013).

5 Health Canada. *Federal Contaminated Site Risk Assessment in Canada, Part I: Guidance on Human Health Preliminary Quantitative Risk Assessment (PQRA)*. Ottawa (2004).

6 U.S. Environmental Protection Agency. *Exposure factors handbook. Office of Research and Development. Washington* (2011).

7 U.S. Environmental Protection Agency. *Risk Assessment Guidance for Superfund (RAGS), Volume I: Human Health Evaluation Manual (Part E, Supplemental Guidance for Dermal Risk Assessment) Interim* (2004).

8 U.S. Environmental Protection Agency. *Cadmium (CAS RN 7440-43-9). Integrated Risk Information System (IRIS)*. (1992) Available at: http://www.epa.gov/iris/subst/0141.htm. (Accessed: 2014)

9 YU, Y. J. *Recognition technique and evaluation method of environmental pollution and health*. (Science Press, 2014).
